# Reliability of the Scale of Barriers for Cardiac Rehabilitation in the Colombian Population

**DOI:** 10.3390/ijerph18084351

**Published:** 2021-04-20

**Authors:** Adriana Marcela Jácome Hortúa, Adriana Angarita-Fonseca, Carmen Juliana Villamizar Jaimes, Rocio del Pilar Martínez Marín, Hugo Celso Dutra de Souza, Tábata de Paula Facioli, Juan Carlos Sánchez-Delgado

**Affiliations:** 1 Grupo de Investigación Fisioterapia Integral, Facultad de Ciencias de la Salud, Universidad de Santander, Bucaramanga, 680003 Santander, Colombia; AD.JACOME@mail.udes.edu.co (A.M.J.H.); rocio.delpilar@udes.edu.co (R.d.P.M.M.); 2 Grupo de Investigación CliniUDES, Grupo de Investigación Fisioterapia Integral, Facultad de Ciencias de la Salud, Universidad de Santander, Bucaramanga, 680003 Santander, Colombia; adriangarita@udes.edu.co; 3 Department of Health Sciences, Université du Québec en Abitibi-Témiscamingue, Rouyn Noranda, QC J9X 5E4, Canada; 4 Centre de Recherche du Centre Hospitalier de l’Université de Montréal, Montreal, QC H2X 0A9, Canada; 5 Profesionales de la Salud, 680003 Bucaramanga, Colombia; innovacionprofesionalsalud@gmail.com; 6 Laboratory of Cardiology, Physiology and Physical Therapy, Ribeirão Preto Medical School, University of São Paulo, Ribeirão Preto 14049-900, Brazil; hugocds@fmrp.usp.br (H.C.D.d.S.); tabatafacioli@usp.br (T.d.P.F.); 7 Grupo de Investigación Ser, Cultura y Movimiento, Facultad de Cultura Física, Deporte y Recreación, Universidad Santo Tomás-Bucaramanga, 680001 Santander, Colombia

**Keywords:** cardiovascular diseases, psychometric testing, treatment adherence and compliance

## Abstract

Cardiac rehabilitation is supported by the highest level of scientific evidence. However, less than 25% of those eligible to participate in a cardiac rehabilitation program initiate it; and of these, 50% drop out prematurely. A modified Spanish Cardiac Rehabilitation Barriers Scale (CRBS) has been translated, culturally adapted and validated in Colombia, however, the reliability remains to be evaluated. This study aimed to determine the internal consistency and test–retest reliability of the CRBS in a Colombian population. In total, 193 patients (67% men, average age = 65 ± 12 years) completed the scale twice, with an average of eight days between applications. Cronbach’s Alpha and intraclass correlation coefficients (ICC) were calculated. The internal consistency of the Colombian version of the CRBS was acceptable (Cronbach’s alpha = 0.84). The ICC of the CRBS was 0.69 (95% CI 0.61–0.76); 0.78 (95% CI 0.71–0.84) when the CRBS was completed by interview; and 0.47 (95% CI 0.21–0.67) when the CRBS was self-reported. The reliability of the interview version of the CRBS was substantial in the Colombian population; however, the reliability of the self-report version was lower. The use of this scale will allow developing strategies to increase participation and adherence to cardiac rehabilitation programs.

## 1. Introduction

According to the World Health Organization (WHO), the leading cause of death worldwide is cardiovascular diseases (CVDs) [1]. CVDs are a group of heart and blood vessel disorders and include coronary heart disease, cerebrovascular disease, rheumatic heart disease, and other conditions [1]. After a cardiovascular event, the survival rate can be increased by 35%, and mortality reduced by 25% if prompt medical attention, such as surgical, pharmacological, and a cardiac rehabilitation program, is given [2,3]. Nevertheless, some studies report that only a range of 7.5–25% of the population eligible to participate in a cardiac rehabilitation program (CRP) initiates it; and of these, 50% dropped out prematurely [2,3]. It has been proven that cardiac rehabilitation is a therapeutic intervention with the highest level of scientific evidence (Class I, level A recommendation) [4,5,6,7].

Different solutions have been proposed to increase adherence to exercise; however, several factors can affect the patient’s commitment to such solutions and alternatives. Factors influencing participation include personal barriers such as age, gender, negative views, reaction to health services, unemployment, and socioeconomic status; and contextual barriers such as distance, transport issues, and lack of family support. Furthermore, the rate of referral to a CRP by medical personnel does not exceed 60%, for which more medical knowledge about the benefits of cardiac rehabilitation is recommended. Likewise, patients should understand the nature of their disease and the most appropriate way to improve their health, which could be achieved through a comprehensive prevention and rehabilitation program. A study conducted in Bucaramanga (Colombia) found that functional status and perceived needs are the most critical barriers to access to CRPs. These barriers may suggest that patients’ lack of awareness about the benefits of cardiac rehabilitation and the difficulties in accepting their health condition may affect participation and adherence to a CRP [8,9,10,11,12,13,14,15].

There are psychometrically validated scales to assess the preferences, obstacles and beliefs towards CRPs [16,17]. Some scales are only applicable to enrolment but not participation; moreover, the criterion validity of the cardiac rehabilitation enrolment obstacles scale was weak [17], as the patient-related obstacles subscale does not differentiate between participants in cardiac rehabilitation and those not enrolled. The Cardiac Rehabilitation Barriers Scale (CRBS) was developed in Canada to assess the patient’s perceived barriers at the patient, provider, and health system levels, affecting their participation and adherence to CRPs [18]. The CRBS discriminates between those who attend a CRP and those who did not and has been validated in 15 languages (e.g., Portuguese, French, Punjabi, Korean, Indonesian, Persian, and Chinese languages) [19,20,21,22,23,24,25,26,27,28]. The English version was psychometrically validated by Shanmugasegaram et al. [18].

The CRBS is made up of four domains or subscales, each related to a group of barriers: perceived needs/health care factors (nine items), logistic factors (five items), conflicts with work schedule/time (three items), and comorbidities/functional status (four items). There is an additional open-ended item asking for other reason(s) for not attending a CRP. A translation and a cross-cultural adaptation of the CRBS was performed among Colombians. As a result, item 18 from the original scale (“I can manage my heart problem on my own”) was eliminated, and item 14 was modified from “travel (e.g., holidays, business, cottage)” to “due to lack of time (for example travel, I am very busy, and/or I have problems with the schedules of the sessions).” The Content Validity Index of the modified Spanish CRBS showed an acceptable score for relevance (0.86) and pertinence (0.88) [21,24].

However, it is necessary to continue evaluating the psychometric properties of the instrument by assessing its reliability, thus justifying its use in the Colombian context. Therefore, the objective of this study was to determine the internal consistency and test–retest reliability of the CRBS in a Colombian population of patients undergoing percutaneous revascularization.

## 2. Materials and Methods

Following a diagnostic test study was conducted, and clinical variables were collected by reviewing the digital medical records of the participants. On the patient’s first visit, we collected data related to sociodemographic and CRP characteristics, and we administered the Spanish CRBS. The Spanish CRBS is composed of 21 items. Items were rated on a five-point Likert-type scale that ranges from 1 = strongly disagree to 5 = strongly agree. Thus, a high score indicates more barriers to participation in CRPs. The scale consists of four subscales: comorbidities/functional status, perceived needs, personal/family problems and conflict with work schedule/time [18,19,20,21,22,23,24,25,26,27,28].

A convenience sample of one hundred and ninety-three subjects aged 18 years and above was analyzed. Subjects participated in a CRP phase II offered by the rehabilitation center “Profesionales de la Salud y CIA” or at the “University Hospital of Bucaramang—Los Comuneros [29]”. Patients referred by a cardiologist and meeting the CRP institutional eligibility requirements were included. Subjects were excluded from the study if they had some relative or absolute contraindication for cardiopulmonary exercise testing or training, were illiterate or had some mental deficiency that limited them from completing the questionnaire. Before signing the informed consent, all patients were informed about the study’s objectives and the confidentiality of the data.

When questionnaires were self-administered, the participants were supervised. The interval between the two applications was one week. It should be noted that in the second evaluation, and the participants did not have access to the survey applied the week before. The information was collected between August 2018 and July 2019.

A sample size calculation of 166 was obtained, based on the intraclass correlation coefficient (ICC) found in the pilot study by Sanchez et al. (ICC: 0.71) [24], with a power of 80%, and an alpha level of 0.05. This calculation was done with the software Stata 13.1, Stata Corp, U.S [30].

## 3. Statistical Analysis

Stata 13.1 [30] was chosen for the statistical analysis, using a confidence level of 0.05. Categorical variables were presented in absolute and relative frequencies. For continuous variables, the mean and standard deviation were calculated. The normal distribution was evaluated using the Skewness/Kurtosis test. The internal consistency was tested with Cronbach’s alpha using the first evaluation; values between 0.7 and 0.95 were considered acceptable [31]. The instrument’s reliability was evaluated through a test–retest procedure using the ICC. Labels proposed by Landis and Koch [32] were used to interpret the reliability: <0 poor; 0–0.20 slight; 0.21–0.40 fair; 0.41–0.60 moderate; 0.61–0.80 substantial, and 0.81–1 almost perfect reliability. Bland and Altman’s methodology [33] was used to determine the agreement between the first and second evaluations.

## 4. Ethical Considerations

The study was approved by the Scientific Technical Committee of University of Santander (approval number 002-CBU).

## 5. Results

Table 1 shows the study population characteristics: 67% were men; the average age was 65 ± 12 years; 90% were from urban areas; 44% were married; 73% belonged to a contributory health insurance regime. The most prevalent diagnosis was acute myocardial infarction (AMI); 69% of the participants attended between 1 and 11 sessions at the time of the interview, and finally, all were under the care of a physical therapist during the rehabilitation process.

Table 2 shows a Cronbach’s Alpha with values greater than 0.80 in all the items of the Spanish CRBS. Likewise, the overall internal consistency of the Spanish CRBS was acceptable with a Cronbach’s Alpha of 0.84.

Table 3 shows the Spanish CRBS test–retest reliability by item, classified as either moderate or substantial.

The reliability analysis by domains showed the following results: health status perception—ICC: 0.63, 95% CI 0.54–0.71; logistic factor—ICC: 0.76, 95% CI 0.70–0.82; work/time conflicts—ICC: 0.67, 95% CI 0.58–0.74; comorbidities/functional status—ICC: 0.62, 95% CI 0.52–0.70; and an overall ICC of 0.69, 95% CI 0.61–0.76. When analyzing reliability according to the survey administration method, an ICC of 0.78, 95% CI 0.71–0.84 was observed when the Spanish CRBS was administered by an interviewer; and 0.47, 95% CI 0.21–0.67 when the Spanish CRBS was self-reported (See Table 4).

## 6. Discussion

The Spanish CRBS has substantial reliability in subjects participating in a phase II CRP. However, the Spanish CRBS reliability decreased to moderate when the scale was self-reported.

A systematic review identifies that personal and contextual factors affect participation in CRP [34]. Gender, age, the presence of comorbidities, employment status, education level, and transportation are examples of personal factors associated with CRP participation. Moreover, the lack of knowledge about the benefits of cardiac rehabilitation among medical staff could reduce the referral rate to a CRP and prevent disseminating relevant information to the patient that could engage them in a CRP. Likewise, inpatient referral is a robust predictor of CRP attendance [35]. However, to obtain a clearer view of the influence of these factors or the barriers that affect attendance and adherence in CRPs, validated instruments are required [9].

Three instruments with acceptable psychometric properties were found [16,17,18]. First, the Australian Cardiac Rehabilitation Enrolment Obstacles scale showed acceptable internal consistency with a Cronbach’s Alpha of 0.89 [17]. The second one, designed by Cooper et al., shows an acceptable internal consistency (Cronbach’s Alpha between 0.70 and 0.79) [16]. Finally, the CRBS [18] has shown a Cronbach’s Alpha between 0.70 and 0.88 and ICCs between 0.64 and 0.78 in different studies [12,18,23]. These results are in line with the ones obtained in this study (Cronbach’s Alpha of 0.84 and an ICC of 0.69).

The psychometric properties of the Cardiac Rehabilitation Enrolment Obstacles scale [17] and the test designed by Cooper A. et al. [16] were evaluated in revascularized subjects with coronary heart disease. Therefore, its utility in other types of patients is unknown. Moreover, the CRBS has been created for all types of subjects demanding a CRP. However, the Brazilian CRBS is the only version that has assessed its psychometric properties in patients with cardiovascular risk factors, coronary heart disease, heart failure, arrhythmias, peripheral arterial disease and chronic obstructive pulmonary disease [20]. Additionally, the CRBS, when compared to the other two scales, is the only one that evaluates the factors influencing participation or adherence to CRPs [18,19,20,21,22,23,24,25,26,27,28].

It is important to specify the time between the application of the scales due to its effect on the reliability results. The time between measurements was three weeks in the Canadian CRBS version, two and a half months in the Brazilian CRBS version, and an average of eight days in our study. This time was established considering that a prolonged period of evolution can change the perception of the barriers to participating in a CRP, and shorter times can lead to a highly erroneous ICC, significantly altering the scale stability [36].

It is not easy to generalize the results obtained across the different studies, primarily because of the differences between sociodemographic characteristics. For example, in Canada, where the scale was created [18], there is a full coverage health system; while in Europe, 19% of 445 CRPs from 37 countries reported that CRPs were paid by the patients [36] and the referral rate ranged between 32.8 and 77% [37]. Additionally, in the Brazilian CRBS study, most of the population had a high schooling level [20]. Furthermore, in the Colombian CRBS study, the sample was obtained from a single region of the country, meaning that the results may not be generalizable to the entire Colombian population. Additionally, our study did not consider patients eligible for cardiac rehabilitation, who did not participate in a CRP.

The CRBS psychometric properties have been evaluated in subjects with average ages between 54 and 67 years, not considering other age groups that can demand cardiac rehabilitation services [19,20,21,22,23,24,25,26,27,28]. On the other hand, the Colombian version showed less reliability when it was self-reported, although in an interviewer’s presence. These results could be explained by the participants’ level of education, taking into account that 44% only had primary education or had no schooling at all. This limitation could be overcome by evaluating the face validity and language adapting of the Spanish CRBS version among Colombians with lower education levels. Another strategy could be the application of the CRBS scale only through an interviewer.

Identifying the barriers to participating in a CRP through a valid and reliable scale in the Colombian population allows health professionals to establish the reasons for low CRP participation, so specific strategies can focus on the real reasons why patients do not attend a CRP. Due to our findings of adequate Spanish CRBS psychometrics properties, we recommend the use of this scale as part of the patient initial evaluation in the cardiac rehabilitation clinical practicums of the Colombian physiotherapy students and in Colombian cardiac rehabilitation services.

Finally, a CRP alternative that can be considered, especially during the COVID-19 pandemic, is cardiac rehabilitation using telemedicine home-based through participants’ electronic devices. Technology will make possible the incorporation of a CRP at home, including exercise routines, education, and patient monitoring several times a week. Thus, patients can overcome the barrier of distance and other barriers that prevent them from participating in in-person programs.

## 7. Conclusions

The Spanish Cardiac Rehabilitation Barriers Scale has a substantial reliability in the Colombian population included in this study. However, its reliability decreases when it is self-reported. Identifying barriers using this scale will allow developing strategies to increase participation and adherence to cardiac rehabilitation programs focused on the real needs of patients.

## Figures and Tables

**Table 1:** Baseline characteristics of the patients.

Variable		*n* = 193	%
Sex	Male	129	67
Age	Median (SD)	65	12
Place of origin	Rural	19	10
	Urban	174	90
Marital status	Single	28	14
	Divorced	22	11
	Married	84	44
	Widow/er	38	20
	Common law	18	9
	NR	3	2
Socio-economic status	Low	138	72
	Medium–high	52	27
	NR	3	1
Health insurance	Subsidized	34	18
	Contributive	142	73
	Special/pre-paid	16	8
	NR	1	1
Education level	None/primary school	85	44
	Middle school	43	22
	Technician	16	8
	Post-graduate	49	26
CR Indication	AMI/ACS	88	46
	Bypass	21	11
	Angioplasty	41	21
	Valvopathy	16	8
	Syncope	2	1
	Other	25	13
Physical disability	No	180	93
	Yes	13	7
Number of cardiac rehabilitation sessions attended	1–11	133	69
	12–23	35	18
	24–36	17	9
	>36	1	1
	NR	7	3
Health professionals involved in CRP	Physiotherapist	193	100
	Psychologist	50	26
	Nutritionist	73	38
	Cardiologist	165	85
	Nurse	77	40
Survey administration method	Self-report	46	22
	Interview	147	75

AMI: acute myocardial infarction; ACS: acute coronary syndrome; CR: cardiac rehabilitation; SD: standard deviation; NR: no response.

**Table 2:** Cardiac Rehabilitation Barriers Scale (CRBS) internal consistency.

**CRBS Item**	**Alpha**
**Health Status Perception**	
(1) …I didn’t know about CR	0.83
(2) …I don’t need CR	0.83
(3) …I already exercise at home or in my community	0.83
(4) …my doctor didn’t feel it was necessary	0.83
(5) …many people with heart problems don’t go to CR and they are fine	0.83
(6) …I can manage on my own	0.83
(7) …I think I was referred but the rehab program didn’t contact me	0.83
(8) …it took too long to get referred and into the program	0.83
(9) …I prefer to take care of my health alone.	0.84
**Logistic Factors**	
(1) Distance	0.83
(2) Cost	0.83
(3) Transportation problems	0.83
(4) Family responsibilities	0.83
(5) Severe weather	0.83
**Work/Time Conflicts**	
(1) Time constraints	0.83
(2) Work responsibilities	0.83
**Functional Status**	
(1) I find exercise tiring or painful	0.83
(2) I don’t have energy	0.83
(3) Other health problems prevent me for going	0.84
(4) I am too old	0.83
**Internal Consistency**	0.84

CI: confidence interval; CR: cardiac rehabilitation; CRBS: Cardiac Rehabilitation Barriers Scale.

**Table 3:** Test–retest reliability of the Cardiac Rehabilitation Barriers Scale.

**CRBS Item**	**ICC**	**95% CI**
**Health Status Perception**		
(1) …I didn’t know about CR	0.61	0.51–0.69
(2) …I don’t need CR	0.38	0.25–0.50
(3) …I already exercise at home or in my community	0.59	0.48–0.67
(4) …my doctor didn’t feel it was necessary	0.46	0.34–0.56
(5) …many people with heart problems don’t go to CR and they are fine	0.39	0.27–0.50
(6) …I can manage on my own	0.48	0.36–0.58
(7) …I think I was referred but the rehab program didn’t contact me	0.45	0.33–0.55
(8) …it took too long to get referred and into the program	0.38	0.26–0.49
(9) …I prefer to take care of my health alone.	0.58	0.48–0.67
**Logistic Factors**		
(1) Distance	0.70	0.62–0.76
(2) Cost	0.70	0.62–0.76
(3) Transportation problems	0.68	0.60–0.75
(4) Family responsibilities	0.55	0.45–0.64
(5) Severe weather	0.61	0.52–0.69
**Work/Time Conflicts**		
(1) Time constraints	0.65	0.57–0.73
(2) Work responsibilities	0.64	0.55–0.71
**Comorbidities/Functional Status**		
(1) I find exercise tiring or painful	0.69	0.61–0.75
(2) I don’t have energy	0.56	0.45–0.65
(3) Other health problems prevent me for going	0.50	0.39–0.60
(4) I am too old	0.60	0.50–0.68

ICC: intraclass correlation coefficient; CI: confidence interval; CRBS: Cardiac Rehabilitation Barriers Scale.

**Table 4:** Reliability of the Cardiac Rehabilitation Barriers Scale by domains and type of survey.

CRBS Item	ICC	95% CI	Interview		Self-Reported	
			ICC	95% CI	ICC	95% CI
Health status perceptions	0.63	0.54–0.71	0.72	0.63–0.79	0.45	0.19–0.66
Logistic factors	0.76	0.70–0.82	0.82	0.76–0.86	0.60	0.38–0.75
Work/time conflict	0.67	0.58–0.74	0.66	0.55–0.74	0.71	0.53–0.82
Comorbidities/FS	0.62	0.52–0.70	0.65	0.55–0.74	0.52	0.27–0.70
Total	0.69	0.61–0.76	0.78	0.71–0.84	0.47	0.21–0.67

FS: functional status; CI: confidence interval; CRBS: Cardiac Rehabilitation Barriers Scale; ICC: intraclass correlation coefficient.

## Data Availability

The data presented in this study are available on request from the corresponding author.
